# P-1437. Racial Disparities in Inpatient Antibiotic Prescribing for Community-Acquired Pneumonia

**DOI:** 10.1093/ofid/ofae631.1611

**Published:** 2025-01-29

**Authors:** Matthew Rock, Keith W Hamilton, Florence Momplaisir, Leigh Cressman, Ebbing Lautenbach, Lauren Dutcher

**Affiliations:** University of Pennsylvania, Philadelphia, Pennsylvania; University of Pennsylvania Perelman School of Medicine, Philadelphia, Pennsylvania; University of Pennsylvania, Philadelphia, Pennsylvania; University of Pennsylvania Perelman School of Medicine, Philadelphia, Pennsylvania; University of Pennsylvania, Philadelphia, Pennsylvania; University of Pennsylvania Perelman School of Medicine, Philadelphia, Pennsylvania

## Abstract

**Background:**

Racial disparities have been reported in antibiotic prescribing for certain inpatient and outpatient conditions, but it remains unknown if antibiotic prescribing for inpatients with community-acquired pneumonia (CAP) have similar patterns despite long-standing guidelines for CAP treatment regimens. We evaluated racial disparities in antibiotic prescribing for inpatients with CAP at three hospitals within a health system.

**Figure:**
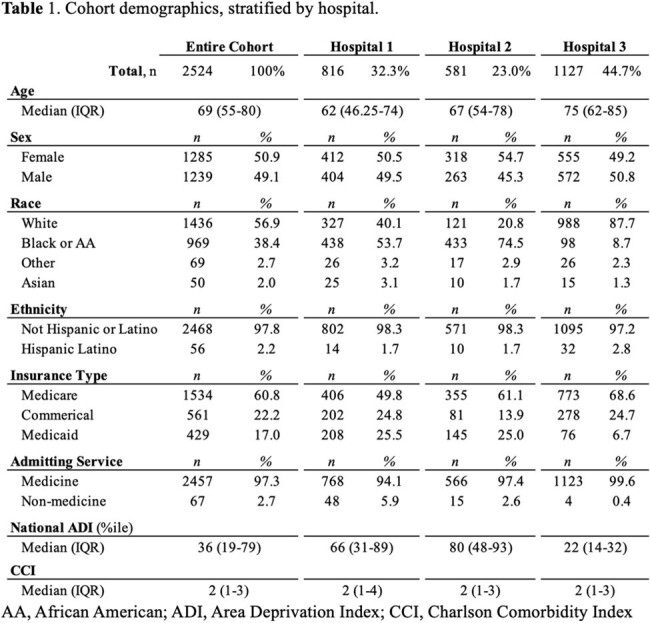

**Methods:**

We conducted a retrospective cohort study with data from the electronic health record using two binary metrics to evaluate differences in the appropriateness of antibiotic selection and duration by race. The cohort included non-ICU inpatients from 1/1/2019-12/31/2021 with a diagnosis of CAP based on ICD-10-CM code who received a systemic antibiotic within 48 hours of admission. We used multivariable logistic regression to determine the association of race with prescribing (selection and duration in separate models), adjusting for variables such as hospital, admitting service, age, sex, Charlson Comorbidity Index (CCI), and area deprivation index.

**Figure:**
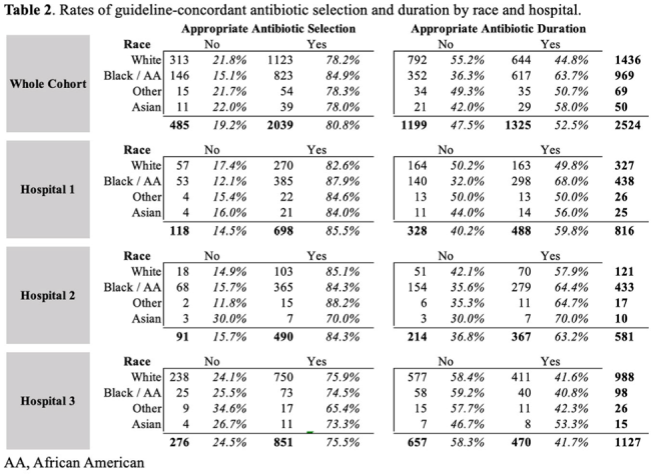

**Results:**

Patient demographics are described in Table 1. Of 2,524 CAP episodes, subjects were prescribed an appropriate empiric antibiotic for an appropriate duration in 42.6% of cases. The rate of appropriate selection was 83%, while the rate of prescribing for the appropriate duration was 53% (Table 2). In the multivariable model, the following were associated with appropriate selection: hospital, lower CCI, and admitting service; race was not associated with antibiotic selection. Black race, hospital, lower CCI, and admitting service were associated with appropriateness of antibiotic duration. (Table 3).

**Figure:**
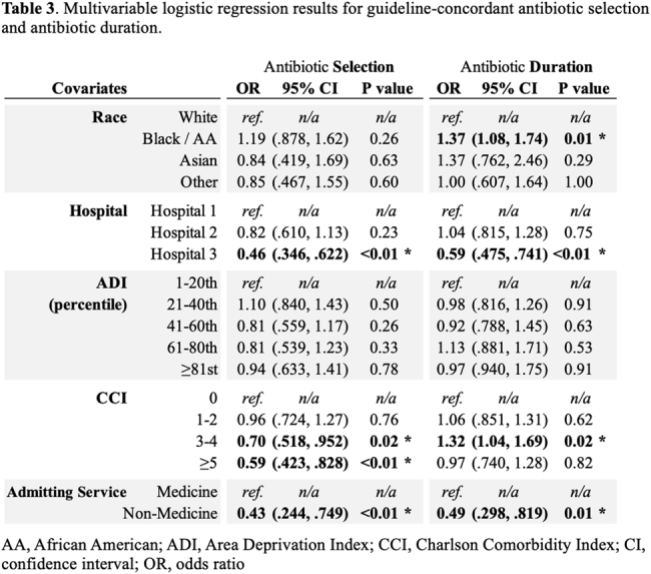

**Conclusion:**

While patient race was not independently associated with the likelihood of appropriate antibiotic selection, it was associated with a higher likelihood of appropriate duration. Importantly, the distribution of patient race differed significantly between hospitals and in both models we found that hospital site was associated with antibiotic prescribing appropriateness. Future research is required to explore the systems-level mechanisms creating these differences in order to ameliorate disparities.

**Disclosures:**

**All Authors**: No reported disclosures

